# Effects of a randomized-controlled and online-supported physical activity intervention on exercise capacity, fatigue and health related quality of life in patients with post-COVID-19 syndrome

**DOI:** 10.1186/s13102-024-00817-5

**Published:** 2024-02-02

**Authors:** Arno Kerling, Sebastian Beyer, Meike Dirks, Michèle Scharbau, Ann-Katrin Hennemann, Alexandra Dopfer-Jablonka, Viktoria Lampe, Jakob Heinrich Wolfgang Salzmann, Uwe Tegtbur, Nora Drick, Isabell Pink, Sven Haufe

**Affiliations:** 1 Clinic for Rehabilitation and Sports Medicine, Hannover Medical School, Carl–Neuberg–Str. 1, 30625 Hannover, Germany; 2 Department of Respiratory Medicine, Hannover Medical School, Hannover, Germany; 3 Clinic for Neurology, Hannover Medical School, Hannover, Germany; 4 Department of Rheumatology and Immunology, Hannover Medical School, Hannover, Germany

**Keywords:** Exercise, Mental health, Physical performance, SARS-CoV-2, Telerehabilitation

## Abstract

**Background:**

The Post-COVID-19 syndrome (PCS), which can occur after acute respiratory syndrome coronavirus 2 infection, leads to restrictions in everyday activity. Our study assessed the impact of an online-guided intervention which intended to facilitate physical activity on the mental and physical capability of PCS patients.

**Methods:**

We randomized 62 patients with PCS (20 male/ 42 female; age: 46 ± 12 years; body mass index: 28.7 ± 6.7 kg/m^2^) with a score ≥ 22 in the fatigue assessment scale (FAS) to a 3-month exercise-focused intervention (IG *n* = 30) or control period (CG *n* = 32). We assessed changes in exercise capacity (bicycle exercise test with measurements of gas exchange), fatigue, markers of health-related quality of life (HrQoL) and mental health.

**Results:**

The FAS score decreased significantly in both study groups (IG: 35.1 ± 7.4 to 31.8 ± 8.5 points; CG: 35.6 ± 7.4 to 32.6 ± 7.5 points, both *p* < 0.01). Exercise capacity did not increase in the CG or IG (within-group changes for IG: peak oxygen uptake: 0.9 ± 2.6 ml/min/kg, *p* = 0.098; peak power output: 6.1 ± 17.8 W, *p* = 0.076) with no significant changes in HrQoL and work ability. Patients with a FAS score at baseline ≥ 35 (severe fatigue) showed no change in exercise capacity with the 3-month intervention whereas the sub-group of patients with FAS < 35 points (moderate fatigue) showed improvements, independent of the study group.

**Conclusions:**

Our 3-month intervention seems appropriate for patients with moderate fatigue, whereas those with more severe fatigue appear to be too restricted with respect to their mental or physical health status to perform exercise at a level which is sufficient to improve markers of physical performance.

**Trial registration:**

German Clinical Trials Register (registration trial number: DRKS00026245) on September 2 2021.

**Supplementary Information:**

The online version contains supplementary material available at 10.1186/s13102-024-00817-5.

## Background

According to the World Health Organization (WHO) Delphi Consensus Method, a Post-COVID-19 syndrome (PCS) is defined as a persistent, recurrent or fluctuating symptomatology which (i) is present later than 12 weeks after an acute infection with severe respiratory syndrome coronavirus 2 (SARS-CoV-2), (ii) lasts for at least two months with (iii) the exclusion of alternative etiologies [1]. It is estimated that PCS occurs in 10–20% of all infected people, affecting not only seriously ill patients but also those with a mild or even an asymptomatic course [2, 3]. In addition to biographical factors (European, middle age, female sex), pre-existing conditions such as arterial hypertension, diabetes mellitus, obesity, bronchial asthma and impaired mental health, as well as criteria specific to coronavirus disease (COVID-19), specific criteria (among others a high number of symptoms, a high viral load and low basal SARS-CoV-2, immunoglobulin G, are the main risk factors for developing PCS [4]. SARS-CoV-2 enters the human body through the binding of its spike glycoprotein to the human angiotensin-converting enzyme 2 (ACE2) receptor [5]. Although the infection usually originates in the respiratory tract, other organ systems such as cardiovascular, gastrointestinal, renal, and central nerve system can also be affected due to the ubiquitous localization of the ACE2 receptors [6].

Currently, there is no evidence-based treatment concept for PCS, even though therapies for some components have been effective for certain sub-groups of patients [7]. The courses of PCS are varied but most of them lead to restrictions in everyday life caused by chronic fatigue, reduced exercise tolerance, dyspnea, neurocognitive problems, muscle pain, sleep disturbances and headache [8]. Fatigue is a leading symptom in PCS and can progress to chronic fatigue syndrome (CFS) if it persists for more than 6 months and is accompanied by post-exertional malaise (PEM) [9].

The aim of our study was to find out whether the impaired physical performance, fatigue and health-related quality of life (HrQoL) could be improved during a 3-month individualized, app-based physical activity intervention adapted to the limited physical capacity of the patients. Our null hypothesis was that the examined outcomes did not change significantly different between the intervention (IG) and control (CG) groups, with peak oxygen uptake (V̇O_2peak_) selected as the main outcome and mental and physical capability as secondary outcomes.

## Methods

### Study design and patients

This was a prospective, randomized, parallel-group, and single-blind (assessor blind) study conducted between September 2021 and December 2022. Volunteers were randomized 1:1 into an IG and a waiting CG using a computer-based list of random numbers generated by the software SPSS. Variable block length with block sizes of 2, 4, and 6, was used to avoid selection bias due to predictability. Study nurses and physicians screening volunteers and assessing the primary outcome at baseline and after 6 months were blinded for the randomization sequence. We recruited eligible patients who consulted the pneumological post-COVID-19 outpatient clinic or the Department of Rheumatology and Immunology of Hannover Medical School. Patients were referred by general practitioners or pneumologists due to persisting symptoms ≥ 3 months after SARS-CoV-2 infection.

According to pre-study defined criteria, we included female and male volunteers aged 18 years or older who reported a continuing impairment of physical or mental health after COVID − 19 (detection by polymerase chain reaction) infection with a fatigue assessment scale (FAS) score ≥ 22 points. Non-inclusion criteria were current participation in another intervention study, clinically relevant acute or chronic infections, pregnancy, preceding surgery less than 8 weeks prior to recruitment, joint replacement that is less than 6 months old, tumor -associated diseases in the last 5 years, or any illnesses or functional impairments which preclude participation in a physical training intervention.

Our randomized clinical trial (RCT) was carried out in accordance with the Declaration of Helsinki, and was registered at German Clinical Trials Register (registration number: DRKS00026245). The institutional ethics review board of Hannover Medical School approved the study (No.9822_BO_S_2021) and written informed consent was obtained prior to the inclusion of patients. All methods were performed in accordance with the relevant guidelines and regulations. This study adhered to CONSORT guidelines [10].

### Primary outcome

The primary outcome of our study was the change in V̇O_2peak_ during an exercise test, recorded at baseline, and after the 3-month intervention period compared with controls. Body weight-normalized values for V̇O_2peak_ and for maximum power output were also expressed as a percentage of age- and sex-adjusted reference values [11, 12]. As V̇O_2_max (defined as maximum volume of oxygen the body can utilize under maximum load conditions and accompanied by V̇O_2_ plateau formation) is often achieved only by competitive athletes or highly motivated subjects, we used V̇O_2peak_ as an alternative (highest oxygen uptake over a 30s interval attained during a particular test).

### Secondary outcomes

Secondary outcomes included the FAS score, HrQoL, severity of depression and anxiety, work ability, body weight, and spirometric parameters. These data were assessed at baseline and after the 3-month intervention period and compared with changes assessed within the control group.

### Assessments

After study inclusion, all patients completed a comprehensive medical evaluation including pulmonary function testing by body plethysmography according to international technical standard [13]. We assessed height and weight in a standardized fashion and estimated fat and fat-free mass with a bioimpedance analysis (InBody 720, Biospace, Seoul, Korea). To determine steps per day, patients received a wearable activity tracker (Forerunner 45, Garmin, Olathe, United States).

For testing parameters of exercise capacity, including the primary outcome (i.e. V̇O_2peak_), patients performed an incremental exercise test using a spirometric system (Oxycon CPX, CareFusion, Würzburg, Germany) on a speed-independent bicycle ergometer (Ergoline P150, Bitz, Germany) with 60 to 70 revolutions per minute. The incremental exercise tests were conducted in an air-conditioned room. To ensure consistent testing conditions, ambient conditions were maintained. To avoid overexerting patients with anticipated low performance, we adopted a testing procedure for the majority of cases based on an individually defined starting load with load increases following evaluation by the investigating physician. With the exception of 6 examinations, the incremental test started with a load of 20 W (W) increasing by 10 W steps every minute and was stopped with the onset of subjective overexertion due to peripheral muscle fatigue and/or pulmonary limitations (4 subjects started with a load of 50 W increasing by 10 W steps every minute, two started with a load of 50 W increasing by 16.67 W steps every minute). Heart rate (12-channel electrocardiogram) and oxygen uptake (breath by breath) were continuously recorded. The subjective perceived exertion was assessed by the Borg scale, with values ranging from 6 to 20 [14]. This scale is validated and used in many languages including the here used German version [15], and based on the assumption that the perception of exertion at maximal voluntary exhaustion is related to an individuals’ heart rate [14].

Self-reported outcomes were recorded via validated questionnaires in German to be completed by patients at home. Fatigue was estimated with the FAS as recommended by the German Society for Pneumology and Respiratory Medicine [16]. Higher numbers refer to more severe fatigue. An FAS score of at least 22 points indicates fatigue, while a score of at least 35 points indicates extreme fatigue [17]. We distributed the authorized version (Hofgrefe Publishing GmbH, Göttingen, Germany) of the short form 36 questionnaire (SF-36) for the estimation of HrQoL [18]. The SF-36 uses eight subscales, each with a scale ranging from 0 to 100, culminating in two summated scales, the mental and physical component score. A higher mental and physical component score corresponds to a better HrQol. We compared the component scores with normative values from the German population [19]. We used the German version of the Hospital Anxiety and Depression Scale (HADS-D), a validated questionnaire to assess the severity of depression and anxiety [20, 21]. Scores for the anxiety and depression subscales range from 0 to 21, higher scores indicating more severe anxiety or depression. Values between 8 and 10 are suggestive for a mood disorder, scores > 10 indicate depressive symptoms and/ or anxiety. To estimate work ability we distributed the work ability index questionnaire [22]. This questionnaire contains seven questions concerning work, work ability and health, resulting in a total score ranging from seven to 49, with higher values representing greater work ability. We estimated daily physical activity characteristics, using the Freiburger activity questionnaire which estimates the total and exercise-related physical activity of adults, both of which are specified as metabolic equivalent of task (MET)-hours per week [23]. All outcome data at baseline and after the intervention were assessed at the Department of Rehabilitation and Sports Medicine at Hannover Medical School. The study intervention was conducted as an online-supported telerehabilitation with the use of the activity tracker (see paragraph “Study Intervention”).

### Study intervention

Patients allocated to the 3-month intervention received an exercise plan recommending 150 min of moderate physical activity per week (60–75% of the maximum heart rate measured during the incremental exercise test). The exercise plan was individually designed by the exercise scientist based on the results of the assessment, personal preferences and needs, as well as the patient’s exercise tolerance. The exercise plan consistently included a defined exercise heart rate, periods of endurance (e.g. cycling, walking, indoor cycling, cross-training, swimming or jogging), strength (e.g. equipment-based training at a gym or home, single-limb strength training, stability training or fitness videos focused on strengthening the body) and recovery (e.g. meditation, stretching exercises, breathing exercises, yoga or relaxation retreats). In addition, once a week, more intense exercise was scheduled in the form of three to ten minutes of stair climbing or one to three minutes of sit-to-stand exercises that allowed patients to exceed their exercise heart rate limit, if tolerated. Patients performed the training independently at home. The control group did not receive any specific instructions and were asked to continue with their current lifestyle and everyday activities.

All patients were provided with Garmin activity trackers to record their exercise, activity intensity, and associated parameters such as sleep duration. The trackers were worn on the non-dominant hand during the study period, connected to the Garmin Connect app, and activity data was stored on the Garmin server. Consent from the patient allowed the exercise scientist and the patient to view the activities through the app. In weeks one, four, and eight of the intervention, scheduled face-to-face exercise consultations were conducted via telephone or video call with an exercise scientist to adjust the exercise plan considering current exercise levels and fatigue severity as needed. The patients’ self-assessment of their previous training session and the corresponding heart rate of the training units was the basis for adapting the training plan during the exercise consultation. The exercise scientist also contacted patients to clarify and discuss possible adjustments when discrepancies from the scheduled training plan were detected. For instance, if the exercise scientist finds that the wearable activity device was not worn, or that activities were not recorded or were recorded incorrectly. Patients were free to contact their exercise scientist by telephone or e-mail with questions or needs at any time.

### Statistical analysis

We did not calculate an a priori sample size due to the lack of appropriate studies in that field at the time. Data were first tested for normal distribution and variance homogeneity with Kolmogorov-Smirnov test and the Levene test, respectively. For all outcomes the analysis was carried out with the per-protocol population, including all cases with complete data at baseline and after the intervention for the primary outcome. For the analysis of the primary and all secondary outcomes, an analysis of covariance model was used with the change in the parameter of interest (3 months-baseline) as the response variable and eta quadrat η² as the effect size. Explanatory variables were sex, the respective parameter at baseline, and the study group (intervention vs. control). To test for within-group differences from baseline to end of intervention, a two-sided Students T-Test or a Wilcoxon-Test with Cohens d as the effect size were used. An exploratory sub-group analysis was performed based on the subdivision of the FAS score into fatigue (< 35 points) and extreme fatigue (≥ 35 points). Within-subgroup differences for changes from baseline to end of intervention in terms of V̇O_2peak_ were tested using a Students T-test or Wilcoxon test. The type-I-error was set to 5% (two-sided). All statistical analyses were performed with IBM SPSS 27 Statistics (IBM Corporation, New York, USA). Unless otherwise stated, values are given as mean ± SD.

## Results

**Fig. 1:**
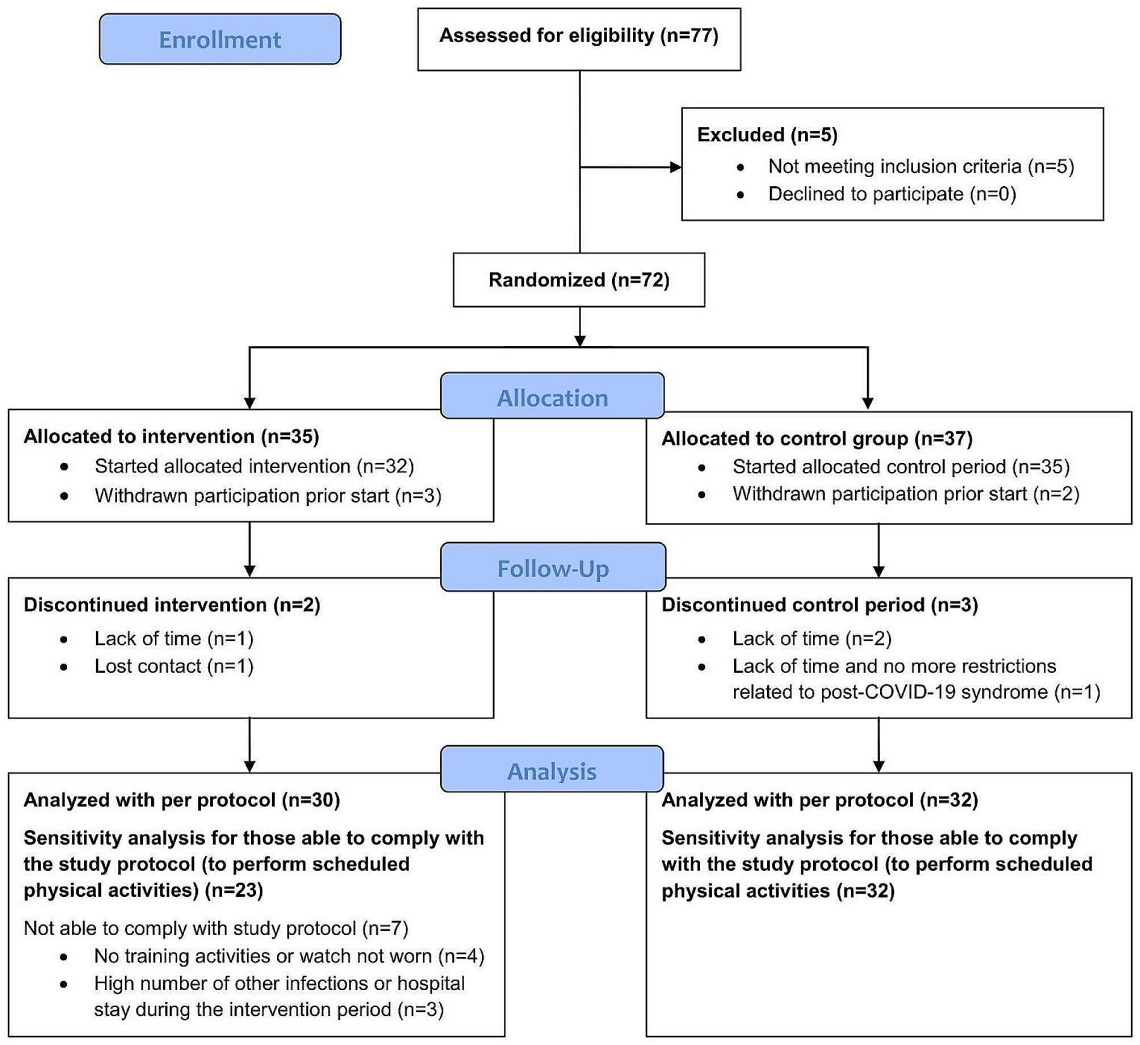
Patient flow during the study. **COVID-19**: Coronavirus disease 2019

Seventy-seven patients were invited for study examinations. Data from 5 patients was excluded from the final analysis. Of the 72 randomized patients, 62 were examined at baseline and after the 3-month study period for the predefined analysis (Fig. 1).

### Patients’ characteristics

The anthropometric and clinical characteristics of recruited patients are shown in Table 1. No significant differences were analyzed between study groups at baseline. On average, the patients’ exercise capacity was below normative values (Table 1), and 34 of 62 patients (55%) presented with extreme fatigue (FAS scale ≥ 35 points).

**Table 1:** Anthropometric and clinical characteristics of patients

	(*n* = 62)	IG (30)*	CG (32)*
female/male (n)	42/ 20	22/ 8	20/ 12
age (yrs)	46.4 ± 11.2	47.1 ± 12.5	46.9 ± 10.1
bodyweight (kg)	84.2 ± 22.5	87.7 ± 27.1	80.7 ± 16.2
BMI (kg/m^2^)	28.7 ± 6.7	29.5 ± 7.5	27.7 ± 5.5
fat mass (%)	31.4 ± 11.5	33.6 ± 11.5	29.4 ± 11.3
FAS (points)	35.2 ± 7.3	35.1 ± 7.4	35.6 ± 7.4
FEV_1_ (l)	3.19 ± 0.83	3.20 ± 0.83	3.18 ± 0.84
FEV_1_ predicted (%)^#^	95 ± 18	98 ± 15	93 ± 20
VC (l)	3.98 ± 1.04	3.93 ± 1.03	4.02 ± 1.07
VC predicted (%)^#^	95 ± 17	96 ± 15	94 ± 18
***Daily physical activity***			
total physical activity (MET-h/wk)	22.4 ± 12.7	21.2 ± 10.7	24.2 ± 11.5
exercise activity (MET-h/wk)	4.7 ± 6.0	3.5 ± 3.9	6.6 ± 7.4
steps per day	7797 ± 3287	7414 ± 2851	8155 ± 3492
***Exercise capacity***			
power output_max_ (watt/kg)	1.64 ± 0.47	1.62 ± 0.61	1.74 ± 0.57
power output_max_ relative to norm value (%)	86 ± 25	84 ± 26	88 ± 24
V̇O_2peak_ (ml/min/kg)	22.4 ± 6.4	21.5 ± 6.1	23.3 ± 6.5
V̇O_2peak_ relative to norm value (%)	72 ± 19	70 ± 19	75 ± 19
***Mental and physical capability***			
work ability index (points)	23.2 ± 8.3	23.6 ± 6.7	23.0 ± 9.6
*Health-related quality of life*			
SF-36 physical component score (points)	35.1 ± 9.2	35.3 ± 8.4	34.9 ± 9.8
SF-36 mental component score (points)	40.5 ± 13.3	40.7 ± 13.9	40.4 ± 13.0
*Anxiety and depression scale*			
depression severity (points)	7.1 ± 4.4	6.7 ± 4.1	7.5 ± 4.8
anxiety severity (points)	7.1 ± 4.8	4.9 ± 0.9	4.7 ± 0.8

Data were mean ± SD

**BMI**: Body mass index. **CG**: Control group. **FAS**: Fatigue assessment scale. **FEV**_**1**_: Forced expiratory volume in one second. **IG**: Intervention group. **MET**: Metabolic equivalent of task. **V̇O**_**2peak**_: Peak oxygen uptake. **SF-36**: Short form 36. **VC**: Vital capacity

Note: * No significant differences for any parameter was found between the IG and the CG. ^#^ according to reference [24]

### Daily physical activity

Questionnaire-estimated overall physical activity increased significantly only for patients in the IG during the 3-month intervention (pre: 21.2 ± 10.7; post: 34.4 ± 22.7 MET-hours/wk, *p* = 0.02, d= − 0.66), but not in the CG (pre: 24.2 ± 11.5, post: 28.4 ± 18.2 MET-hours/wk; *p* = 0.43, d= − 0.21) with no differences between groups over time (*p* = 0.34). Exercise-related physical activities or steps per day did not change significantly for either study group. Individual training data for the IG are given at supplementary Table S1.

### Changes for primary and key secondary outcomes

**Fig. 2:**
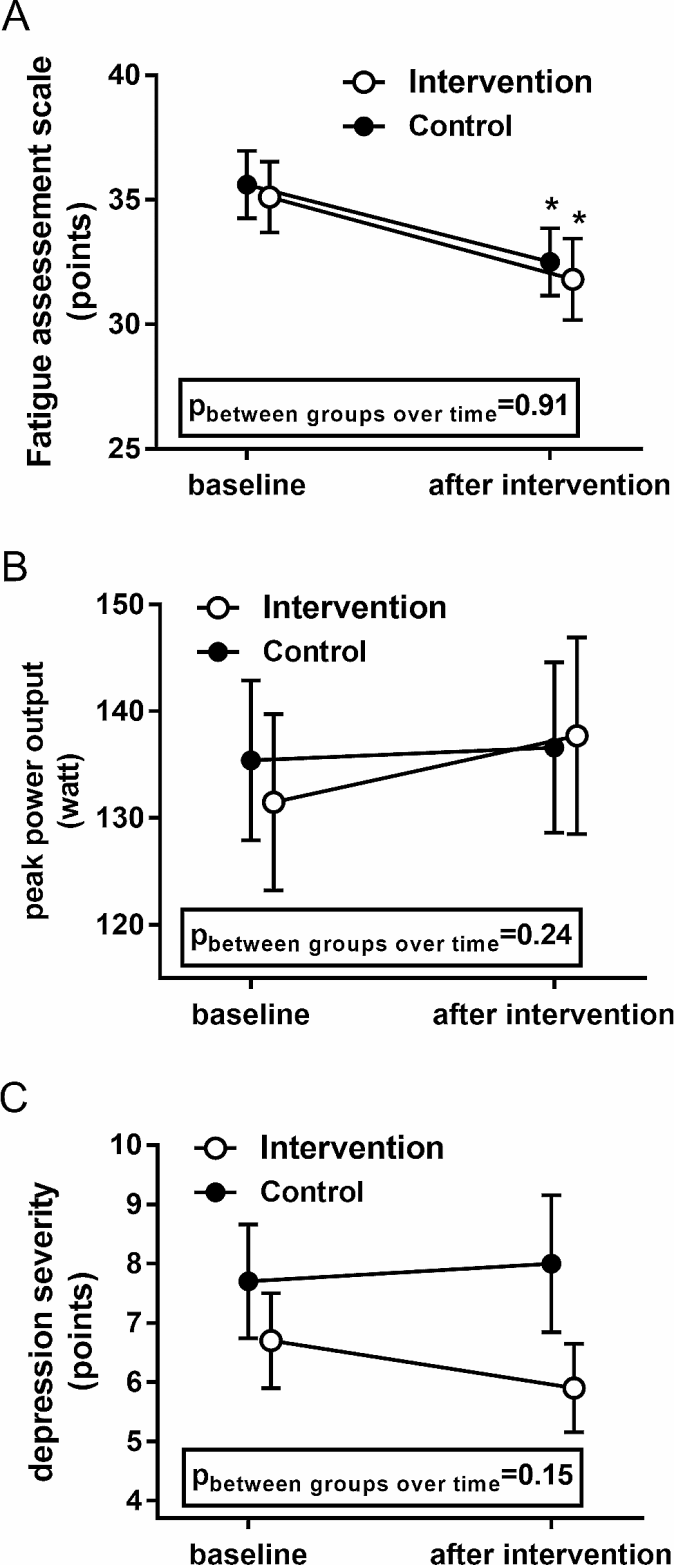
Effects of the 3-month study on fatigue (A), exercise capacity (B) and depression severity (C) of the intervention group (*n* = 30) and the control group (*n* = 32). Data were mean (SEM). ^*^Significant difference between baseline and after intervention within-group as analysed with a Student´s T-Test for paired samples. The framed *p*-value gives the significance level for the difference between groups over time as assessed with an analysis of covariance adjusted for age, sex and the respective baseline value of the tested parameter

After 3 months, V̇O_2peak_ did not change in either group (IG: 21.6 ± 6.3 to 22.5 ± 7.4 ml/min/kg, *p* = 0.10, d= − 0.32; CG: 23.3 ± 6.7 to 23.7 ± 7.2 ml/min/kg, *p* = 0.66, d= − 0.14) (see also Table 2). The FAS score decreased in both the IG (35.1 ± 7.4 to 31.8 ± 8.5 points, d = 0.55) and the CG (35.6 ± 7.4 to 32.6 ± 7.5 points, d = 0.51) (both *p* < 0.01, Fig. 2A). Peak power output during exercise tests did not increase in the IG (131.6 ± 43.8 to 137.7 ± 48.8 W, *p* = 0.076, d= − 0.34, or in the CG (135.4 ± 41.7 to 136.6 ± 44.6 W, *p* = 0.45, d= − 0.08) (Fig. 2B).V Depression severity did not change significantly in the IG (6.7 ± 4.1 to 6.0 ± 3.8 points, *p* = 0.15, d = 0.31) or in the CG (7.7 ± 5.0 to 8.0 ± 5.9 points, *p* = 0.67, d= − 0.08). (Fig. 2C; Table 2). No significant change was observed for any of these parameters between the study groups over time (for changes within groups over time see Table 2).

**Table 2:** Changes in outcome parameters for the intervention (IG) and control (CG) groups

	IG (*n* = 30)		CG (*n* = 32)			
	change from baseline	cohens d	change from baseline	cohens d	mean difference between groups over time [CI95%]	eta quadrat η²
FAS (points)	– 3.3 ± 5.8	0.55	– 3.0 ± 6.3	0.51	0.3 [– 2.6; 3.9]	0.00
V̇O_2peak_ (ml/min/kg)	0.9 ± 2.6	– 0.32	0.3 ± 3.4	– 0.14	– 0.6 [– 1.8; 0.8]	0.01
V̇O_2peak_ (%)	4.0 ± 9.2	-0.44	1.2 ± 8.0	-0.14	2.88 [– 1.78; 7.53]	0.03
*Anxiety and depression scale*						
depression severity (points)	– 0.7 ± 2.4	0.31	0.3 ± 3.5	– 0.09	1.0 [– 0.7; 2.8]	0.03
anxiety severity (points)	0.0 ± 2.4	– 0.02	0.2 ± 2.6	– 0.06	0.2 [– 1.4; 1.6]	0.00
*Health-related quality of life*						
physical component score (points)	2.2 ± 5.7	– 0.38	3.4 ± 7.9	– 0.43	1.2 [– 2.7; 5.1]	0.01
mental component score (points)	2.9 ± 9.2	– 0.31	– 0.1 ± 10.2	– 0.01	– 3.0 [– 8.5; 2.5]	0.02
work ability index (points)	– 0.3 ± 5.7	0.05	0.7 ± 3.5	– 0.19	1.0 [– 1.9; 3.8]	0.01
FEV_1_ (l)	0.03 ± 0.22	– 0.16	– 0.02 ± 0.23	0.08	– 0.05 [– 0.18; 0.07]	0.01
FEV_1_ predicted (%)^#^	1.1 ± 6.3	-0.17	-0.6 ± 6.9	0.09	1.69 [– 2.00; 5.39]	0.02
VC (l)	0.04 ± 0.28	– 0.13	0.04 ± 0.27	– 0.15	0.00 [– 0.15; 0.16]	0.00
VC predicted (%)^#^	0.7 ± 6.5	-0.10	0.8 ± 6.4	-0.12	-0.08 [– 3.69; 3.52]	0.00

**FAS**: Fatigue assessment scale. **FEV**_**1**_: Forced expiratory volume in one second. **VC**: Vital capacity. **V̇O**_**2peak**_: Peak oxygen uptake

**Notes**:

Changes from baseline to after 3-month intervention were analyzed with *Student’s* T-Test for paired samples with data shown as mean ± SD

Differences between groups (IG versus CG) were analyzed by an analysis of variance and shown as mean difference with 95% confidence intervals (CI95%)

We detected no significant differences within groups or between groups over time. ^#^ according to reference [24]

### Health-related quality of life and work ability

The mental component score and the physical component score for HrQoL were below that predicted in relation to normative values (83.4 ± 26.9%; 67.7 ± 16.3%, respectively). After 3 months, the physical and mental component score increased with no significant differences between study groups (Table 2). Work ability did not change in either group with the intervention (Table 2).

**Fig. 3:**
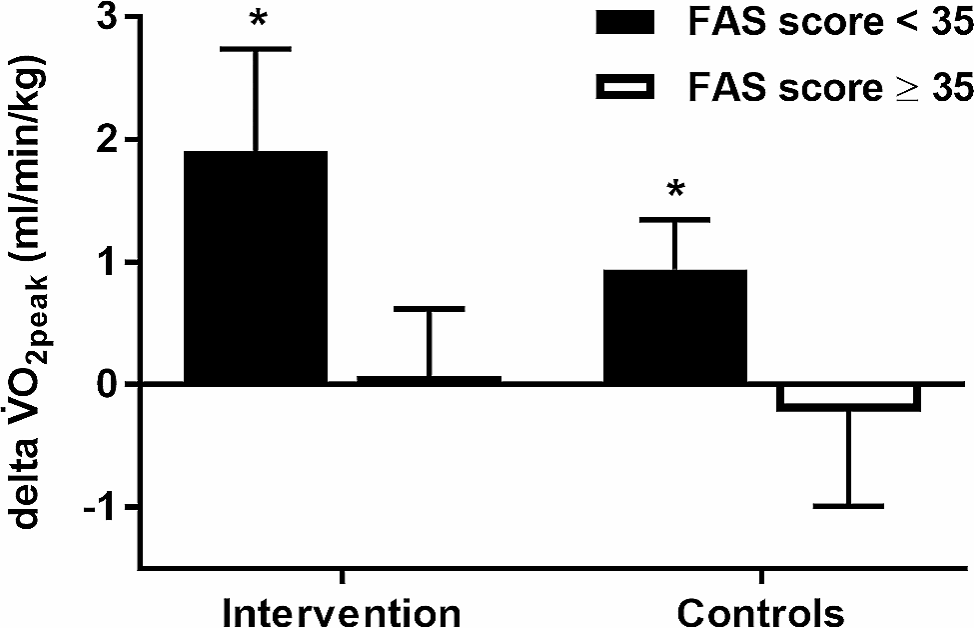
Effects of the 3-month intervention on peak oxygen uptake (V̇O_2peak_) for patients in the intervention (*n* = 30) and control (*n* = 32) groups assembled into those with a fatigue assessment scale (FAS) score below and above 35 points. ^*^p < 0.05 for the delta V̇O_2peak_ (change of V̇O_2peak_) within the specific sub-group as analysed with a Student´s T-Test for paired samples. Data were mean (SEM)

We sub-grouped our cohort in patients below and above a FAS of 35 points at baseline. Those patients with a FAS ≥ 35 showed no change in exercise capacity after the 3-month intervention whereas the sub-group of patients with FAS < 35 points did, independent of whether they were in the IG or CG (Fig. 3).

## Discussion

We observed a heterogeneous response to the 3-month online-supported physical activity intervention. We could not identify significant improvements for exercise capacity, HrQoL or fatigue severity in our study sample.

Before starting the intervention, our patients showed reduced exercise capacity and HrQol which is a common finding in patients suffering from PCS [25, 26]. In addition to deconditioning, a number of other causes, such as autonomic dysfunction or muscular impairments, may also be causal factors in evoking the exertional intolerance [25, 26]. Principally, exercise and pulmonary rehabilitation are promising therapeutic strategies for physical and psychological symptoms, which have positive effects on cardiopulmonary fitness, strength, fatigue and depression, both in the acute phase and in the further course of the disease [27–40]. In severely affected patients with acute respiratory distress syndrome caused by COVID-19, a significant improvement in dyspnea could be achieved after 3 months of exercise intervention in comparison to standard physiotherapy [41]. In non-hospitalized patients with a mild course of disease, supervised training produced better results in terms of HrQol, fatigue and depression and better physical performance, compared with training according to the WHO protocol [42, 43].

In our RCT, with the exception of a significant increase in the questionnaire-estimated overall physical activity in the IG, no significant improvements in parameters of physical performance and HrQol were shown between study groups, regardless of whether or not training was possible according to the study protocol. The minimum clinically important difference (MCID) for a V̇O_2peak_ change (i.e. primary outcome) is considered 1.0 ml/min/kg favoring the intervention group [44]. Therefore the obtained change in exercise capacity after the 3-month intervention is not only non-significant between study groups but also did not reach the MCID for V̇O_2peak_ in our studied patients. We assume that the training intensities that were achievable across all recruited patients were too low to attain an improvement in exercise capacity. RCT’s investigating therapeutic exercise interventions in PCS so far [25, 26] were mostly performed in patients with mild or moderate symptoms, therefore it is likely that the lack of improvement is due to differences in disease severity and not a result of the training method chosen, since telerehabilitation and app-based training have been shown to be effective and helpful in increasing cardiopulmonary performance and HrQol [45, 46].

In terms of the feasibility of an exercise program and the physical activity performed, our study had a very heterogeneous patient population and structured exercise/physical activity was not possible for all patients due to impairment caused by fatigue. Taking into account that exercise is contraindicated in extreme fatigue [7] and in order to prevent PEM and the occurrence of a crash, an individualized exercise program, adapted to the patient’s individual performance, was implemented according to the WHO recommendations [47]. Fatigue, depressive symptoms and deteriorated HrQoL often occur together in PCS [48]. The improvement of the FAS in both groups independent of the implementation of training is indicative of a healing tendency over time. However, our data also show a dependency of the improvement of V̇O_2peak_ and FAS in relation to the FAS baseline value, i.e. the higher the FAS was, the lower the improvement was. We assume that with increasing fatigue, physical exertion becomes less feasible and only a relatively low level of physical activity is possible. Patients with severe fatigue did not show any improvement in performance, whereby their initial level could at least be maintained. Regardless of the intervention, both groups with an FAS < 35 showed a significant improvement in physical performance. In addition, recovery seems to be delayed in patients with severe fatigue. This is an important aspect because different patient groups may need different therapeutic strategies depending on the severity of fatigue symptoms. Fowler-Davis et al. [48] analyzed several different and complementary interventions in terms of their effectiveness on FAS showing benefits for physical activity, which could be more suitable for less severe courses of fatigue.

### Limitations

One limitation in the planning of the study was an overestimation of the resilience of the patients and, despite an individualized training strategy, that the patients were often only able to perform basic movements such as walking or climbing stairs. We did not perform an *a priori* sample size calculation due to the lack of appropriate studies in that field at the time. A *post hoc* power analysis revealed that the statistical power achieved for the between-group difference in V̇O_2peak_ was 12%. With a given statistical power of 80%, a sample size of 810 patients would have been necessary to detect a significant between-group difference over time in V̇O_2peak_ with a two-sided α-error of 0.05. The weak statistical power obtained supports the conclusion that the conducted intervention was not effective in this heterogeneous sample of patients with PCS with significant difficulties in training participation. Therefore, the feasibility of a physical activity intervention for improving PCS-associated symptoms likely requires a multidisciplinary and individually focused approach to improve mental and physical wellbeing in these patients. An extension of the intervention period may also have had an effect on the symptoms due to the subliminal training stimuli. In addition, the study was performed in a German, mainly European population and, therefore, may not have sufficient generalizability to other non-western populations or ethnicities.

## Conclusions

In contrast to many other diseases, structured exercise/physical activity is difficult to perform for patients with PCS due to the pre-existing fatigue. Contrary to our hypothesis, we did not observe effects in exercise performance or mental and physical capability in PCS patients. However, in order to counteract a further deterioration in physical performance, movement and physical activity should be integrated into patients’ everyday life, depending on individual resilience. Our exploratory results suggest that further studies should focus on whether different treatment strategies may need to be prioritized depending on the severity of the fatigue.

### Electronic supplementary material

Below is the link to the electronic supplementary material.

Supplementary Material 1

## Data Availability

The data used to support the findings of this study are available from the corresponding author upon request. The data sets generated and analyzed during the current study are available.
