# Supplementary material for: Effects of a randomized-controlled and online-supported physical activity intervention on exercise capacity, fatigue and health related quality of life in patients with post-COVID-19 syndrome
Source: BMC Sports Sci Med Rehabil. 2024 Feb 2;16:33. doi: 10.1186/s13102-024-00817-5 (PMC10835885; doi:10.1186/s13102-024-00817-5)
Supplement: Supplementary file 1 — Supplementary Material 1 [file 13102_2024_817_MOESM1_ESM.docx]

| **Supplementary Table S1. Individual overview about performed endurance and strength activities during the 3-month intervention for the intervention group.** | | | | | | | |
| --- | --- | --- | --- | --- | --- | --- | --- |
| **ID** | **Heart rate limit  (bpm)** | **Endurance activity** | | | **Strength activity** | | |
|  |  | **Overall number of activities** | **Duration per activity (min)** | **Heart rate per activity (bpm)** | **Overall number of activities** | **Duration per activity (min)** | **Heart rate per activity (bpm)** |
| PC01 | 140 | 45 | 30±34 | 107±7 | 25 | 35±20 | 95±20 |
| PC02 | 135 | 5 | 35±5 | 156±11 | 0 |  |  |
| PC03 | 135 | 39 | 47±25 | 108±16 | 18 | 36±17 | 98±15 |
| PC04 | 110 | 34 | 38±14 | 92±8 | 3 | 34±2 | 99±6 |
| PC10 | 130 | 36 | 42±28 | 112±17 | 8 | 33±19 | 103±4 |
| PC12 | 130 | 103 | 31±17 | 109±9 | 25 | 14±18 | 117±9 |
| PC15 | 105 | 97 | 29±29 | 82±11 | 2 | 20±10 | 64 |
| PC17 | 115 | 57 | 39±19 | 103±9 | 44 | 14±3 | 82±8 |
| PC18 | 120 | 184 | 19±14 | 100±13 | 181 | 5±6 | 111±8 |
| PC20 | 85 | 1 | 25 | 63 | 0 |  |  |
| PC22 | 130 | 27 | 37±29 | 118±8 | 10 | 16±16 | 97±14 |
| PC23 | 150 | 60 | 48±31 | 126±19 | 15 | 49±14 | 100±15 |
| PC25 | 140 | 25 | 38±20 | 132±8 | 44 | 56±13 | 127±11 |
| PC30 | 140 | 133 | 17±11 | 118±15 | 58 | 98±16 | 123±13 |
| PC33 | 110 | 150 | 36±19 | 87±12 | 6 | 30±10 | 75±7 |
| PC35 | 140 | 73 | 50±40 | 104±11 | 7 | 22±17 | 123±18 |
| PC36 | 120 | 68 | 30±24 | 105±10 | 13 | 22±17 | 95±9 |
| PC45 | 130 | 73 | 28±16 | 112±13 | 31 | 41±18 | 104±10 |
| PC46 | 130 | 159 | 38±17 | 99±25 | 115 | 20±8 | 92±10 |
| PC51 | 115 | 44 | 33±14 | 100±13 | 5 | 12 | 95±4 |
| PC52 | 130 | 94 | 42±22 | 100±9 | 52 | 5±8 | 114±13 |
| PC53 | 125 | 36 | 22±22 | 108±16 | 7 | 26±4 | 84±6 |
| PC56 | 110 | 131 | 20±8 | 93±8 | 10 | 6 | 104±7 |
| PC57 | 105 | 105 | 36±19 | 96±11 | 23 | 28±16 | 83±22 |
| PC58 | 130 | 80 | 36±20 | 107±15 | 17 | 36±21 | 100±8 |
| PC63 | 115 | 86 | 35±21 | 106±9 | 24 | 23±14 | 96±11 |
| PC68 | 130 | 9 | 17±23 | 91±15 | 18 | 15±8 | 102±11 |
| PC69 | 135 | 51 | 31±14 | 110±27 | 91 | 21±13 | 84±11 |
| PC71 | 125 | 61 | 82±55 | 96±8 | 66 | 37±28 | 89±10 |
| PC74 | 125 | 26 | 37±37 | 108±18 | 9 | 26±24 | 99±7 |
|  |  | **Endurance activities** | | | **Strength activities** | | |
|  |  | **Mean number of activities per week** | **Mean duration per week (min)** | **Mean heart rate per activity (bpm)** | **Mean number of activities per week** | **Mean duration per week (min)** | **Mean heart rate per activity (bpm)** |
|  |  | 5,8 | 195,4 | 104,9 | 2,6 | 72,2 | 98,4 |
|  |  | 3,9 | 124,9 | 16,0 | 3,2 | 97,7 | 14,3 |
| All manually started and recorded physical activities from the wearable activity tracker worn by patients during the 3-month intervention period were transferred to the Garmin Connect app. The individual upper heart rate limit for exercise activities was defined by the supervising physician after the incremental exercise test and physical examination of the patient. Endurance activities included all everyday activities like walking, cycling or stair stepping. Strength activities included resistance training activities as well as stretching and functional gymnastics.  Average data are mean (SD). It should be noted that the given training data might differ from the actual number due to reliance on correct entries and compliance to record any performed training session. | | | | | | | |
